# *De novo* Transcriptome Sequencing Reveals a Considerable Bias in the Incidence of Simple Sequence Repeats towards the Downstream of ‘Pre-miRNAs’ of Black Pepper

**DOI:** 10.1371/journal.pone.0056694

**Published:** 2013-03-04

**Authors:** Nisha Joy, Srinivasan Asha, Vijayan Mallika, Eppurathu Vasudevan Soniya

**Affiliations:** Plant Molecular Biology, Rajiv Gandhi Center for Biotechnology, Thiruvananthapuram, Kerala, India; Centro de Investigación y de Estudios Avanzados del IPN, Mexico

## Abstract

Next generation sequencing has an advantageon transformational development of species with limited available sequence data as it helps to decode the genome and transcriptome. We carried out the *de novo* sequencing using illuminaHiSeq™ 2000 to generate the first leaf transcriptome of black pepper (*Piper nigrum* L.), an important spice variety native to South India and also grown in other tropical regions. Despite the economic and biochemical importance of pepper, a scientifically rigorous study at the molecular level is far from complete due to lack of sufficient sequence information and cytological complexity of its genome. The 55 million raw reads obtained, when assembled using Trinity program generated 2,23,386 contigs and 1,28,157 unigenes. Reports suggest that the repeat-rich genomic regions give rise to small non-coding functional RNAs. MicroRNAs (miRNAs) are the most abundant type of non-coding regulatory RNAs. In spite of the widespread research on miRNAs, little is known about the hair-pin precursors of miRNAs bearing Simple Sequence Repeats (SSRs). We used the array of transcripts generated, for the *in silico* prediction and detection of ‘43 pre-miRNA candidates bearing different types of SSR motifs’. The analysis identified 3913 different types of SSR motifs with an average of one SSR per 3.04 MB of thetranscriptome. About 0.033% of the transcriptome constituted ‘pre-miRNA candidates bearing SSRs’. The abundance, type and distribution of SSR motifs studied across the hair-pin miRNA precursors, showed a significant bias in the position of SSRs towards the downstream of predicted ‘pre-miRNA candidates’. The catalogue of transcripts identified, together with the demonstration of reliable existence of SSRs in the miRNA precursors, permits future opportunities for understanding the genetic mechanism of black pepper and likely functions of ‘tandem repeats’ in miRNAs.

## Introduction

Generation of a full complement of sequences transcribed in a cell isa reliable tool that enables to discover, profile and quantify genes. Unlike traditional sequencing approaches, transcriptome sequencing rapidly generate large datasets and require relatively shorter time and labor [1]. Transcriptome sequencing serves as an efficient platform in species with very less sequence information to rapidly expose an array of resourceful genes in a single experiment. It also helps to trace out the function of newly identified miRNAs having no significant homologs. In this study, we analysed black pepper (*Piper nigrum* L.), the ‘King of Spices’, which is an important member in the family *Piperaceae* and cultivated for its green and dried fruits. As the centre of origin of Black pepperis Western Ghats of South India, there exists a rich diversity among its cultivars. Majority of the studies conducted on black pepper are confined to biochemical characteristics as this contributes significantly to the taste or ‘spicy’ qualities, especially ‘piperine’ (1-piperoylpiperidine) which is its major alkaloid. Phytochemical and pharmacological studieshave identified anti-inflammatory, analgesic, anticonvulsant, anti-ulcer, antioxidant, cytoprotective and anti-depressant effects [2] of piperine which is of immense interests to researchers. Reports demonstrates that piperine in combination with curcumin can act as potential cancer preventive agents [3]. Except for studies focusing on limited aspects of morphological and biochemical characteristics [4]; molecular markers based assays like AFLP, SSR [5], [6], and *in vitro* cultures [2], [7], [8], corresponding basic or applied research at the genomic level has not been undertaken in the case of black pepper. In spite of the commercial interest and diverse use, very few efforts have been initiated to elucidate either its transcriptome or genome sequence information. *P. nigrum* with 2n = 52 is a tetraploid, predominantly self-pollinated, propagated by stem cuttings with a genome size of approximately 6.68952 Gbp (1C _(mean)_ = 1.71 pg) [9] (http://www.kew.org/cvalues/. Accessed 2013 Jan 17). Altogether 134 sequence count is available in the public domain as of 2011. Hence we are interested to overcome this lacuna of a genomic dictionary in black pepper by applying the next generation illuminaHiSeq™ 2000 sequencing – a rapid, effective, reproducible and high resolution technique, which is demonstrated in the first part.

Microsatellites also known as ‘Short Tandem Repeats’ (STRs) or ‘Simple Sequence Repeats’ (SSRs) are short (1–5 bp), tandem repeated DNA sequences that are believed to have originated from either *de novo* genesis or adoptive genesis [10]. Errors during recombination, unequal crossing over and polymerase slippage during DNA replication or repair, all contribute to the higher mutation rate of microsatellites ranging from 10^−2^ to 10^−6^ nucleotides per locus per generation, when compared to other parts of the genome [11]. Microsatellites are abundant and randomly interspersed in eukaryotic genomes [12], including both coding and non-coding regions of the genome. The relative abundance of different microsatellite motifs varies considerably; (CA)n motif is suggested as the most frequent repeat in humans and many mammals [13], [14], [15], whereas (AT)n motif is most abundant in plant genomes. Till recently SSRs were considered as ‘junk DNA’ and were utilized as genetic markers for fingerprinting studies. Later rapid accumulation of reports highlighting the direct effect during ‘change in the number of SSR motifs in transcripts’, brings the need for understanding the relevance of SSRs in non-coding genomic regions. The significant contribution of repetitive regions in genomic sequences have been well documented in previous reports which suggests that repeat-rich sequences can give birth to small non-coding functional RNAs like heterochromatic small RNAs (hcRNAs) and piwi-interacting RNAs (piRNAs) including its sub type - the repeat-associated small interfering RNAs (rasiRNAs) [16]. Among the non-coding functional regulatory RNAs, the most abundant type is the microRNAs (miRNAs). The biogenesis of miRNAs occur from primary miRNA transcripts known as ‘pri-miRNAs’ which will adopt a stem-loop secondary structure known as the ‘pre-miRNAs’, from which a specific mature 21-nucleotide duplex is excised by a RNase-III-type enzyme Dicer endonuclease. After the processing by Dicer, the miRNAs emerge as siRNA-duplex-like structures, but only one strand, the mature miRNA is predominantly incorporated into the Ago effector complexes. The discarded RNA strand is referred to as miRNA* and is finally degraded [17], [18]. In plants, ‘miRNAs’ of 18–24 nt in length are considered as the key regulators of post transcriptional gene silencing (PTGS) [19]. They are involved in wide range of plant development processes like leaf morphogenesis and polarity [20], [21], floral differentiation and development [22], root initiation and development [23], [24], vascular development [25], [21] and vegetative phase change [26].

Despite several studies, very little is known about hair-pin precursors of miRNAs having SSRs in their sequences. The number of SSRs per pre-miRNA on average ranges from 4.1 for viruses to 13.5 for Mycetozoa when analysed across 87 species includingArthropoda, Nematoda, Platyhelminthes, Urochordata, Vertebrata, Mycetozoa, Protistae, Viridiplantae, and Viruses [27]. Our previous survey [28] across transcribed microsatellites in black pepper identified a miRNA candidate with distinct putative functions related to growth and the candidate was noticeably derived from its hair-pin precursor bearing (CT) dinucleotide repeats. Considering these facts, the transcriptome data generated in the initial part of the study was utilized in such a way so as to segregate the ‘pre-miRNA candidates’. These candidates were further studied for the occurrence, type and distribution of different types of SSR motifs in their sequences. We reasoned that the study of hair-pin precursors of regulatory miRNAs with SSR motifs will provide (a) a good platform for further investigation of possible functions of SSRs (b) valid comparison with hair-pin precursor sequences of well studied species like *Arabidopsis thaliana*.

## Materials and Methods

### Plant Material

About 1 g of tender leaves collected from potted black pepper plant (variety –Panniyur 1) maintained in the green house, was used for total RNA isolation.

### RNA Isolation, cDNA Preparation and Sequencing

Total RNA was isolated using mirVana™ miRNA Isolation Kit (Ambion) according to manufacturer’s instructions. RNA quality was verified using Agilent 2100 and RNA Integrity number (RIN) value was checked before proceeding further. The RNA was quantified using Nanodrop analysis (recommended: A_260/280_ = 1.8 – 2.2; A_260/230_≥2.0; concentration ≥20 *µ*g i.e. 0.4 *µ*g/*µ*L). RNA was subjected to *DNase* treatment using TURBO DNA-*free*™ Kit (Ambion), followed by acid phenol chloroform extraction and ethanol precipitation. The cDNA library preparation and illumina sequencing was performed by Beijing Genomics Institute - HongKong Co. Ltd as per manufacturer’s protocol (Illumina, San Diego, CA). Briefly, isolation of poly (A) mRNA was done using beads with oligo (dT) and the addition of fragmentation buffer for interrupting mRNA into short fragments (200 – 700 nt) avoided priming bias during the synthesis of cDNA using random hexamer-primers. The short fragments were further purified using QiaQuick PCR extraction kit and resolved with EB buffer for ligation with illumina Paired-end adapters. This was followed by size selection, PCR amplification and illumina sequencing.

### Pipeline of Bioinformatic Analysis for Unigene Annotations

**Figure 1:**
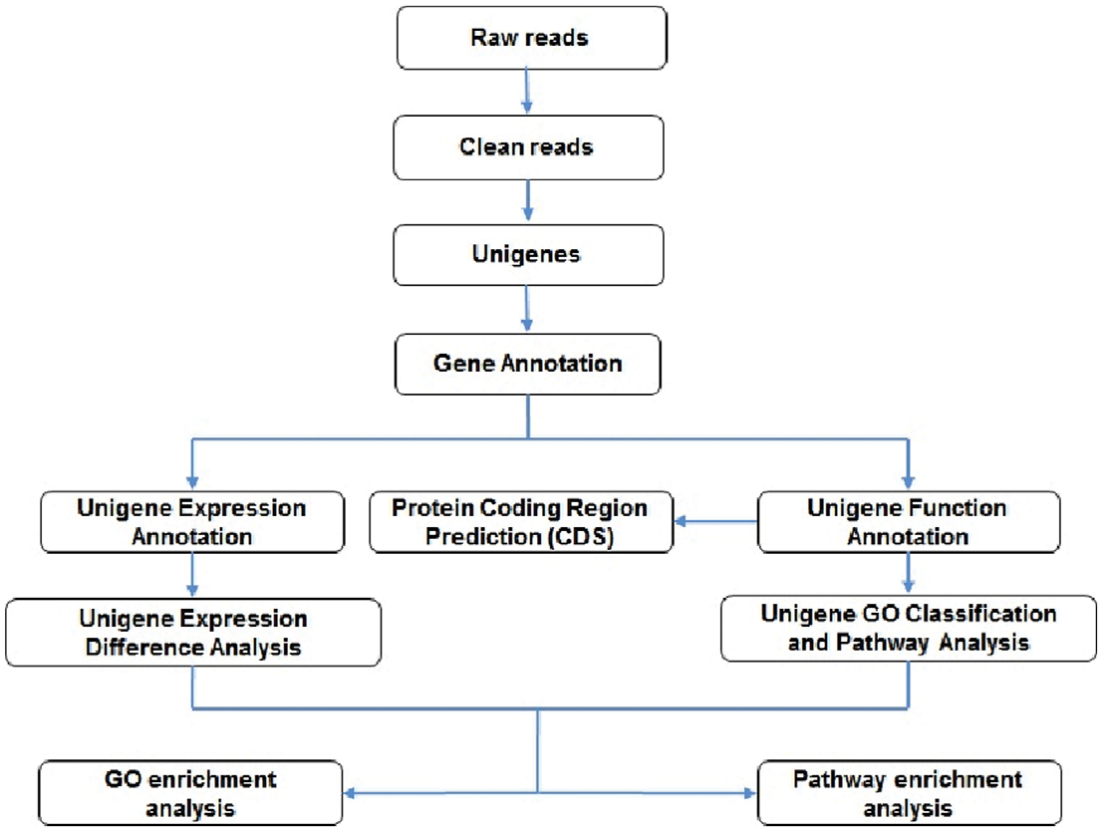
Bioinformatic pipeline followed for annotation of unigenes.

The output of raw reads from sequencer was subjected to stringent filtering conditions like: Removed (a) reads with adaptors; (b) reads with unknown nucleotides larger than 5% and (c) reads with low quality. The clean reads were assembled using short read *de novo* assembler program – Trinity [29] into contigs, scaffolds and finally unigenes. Further annotation of unigenes provided information on its expression levels and function. The expression levels of unigenes were calculated using Reads Per kb per Million reads (RPKM) method [30]. The formula for calculating RPKM is : RPKM value for gene A = (1000000*C)/N*L*1000), where C is the number of reads that uniquely aligned to gene A, N is the total number of reads that uniquely aligned to all genes, L is the number of bases on gene A. Functional annotations were carried out using BLASTx program against protein databases like NCBI non-redundant (Nr), Swiss-Prot, the Kyoto Encyclopedia of Genes and Genomes (KEGG) pathway, Cluster of Orthologous Groups (COG) and Gene Ontology (GO) with nr annotation using Blast2GO program [31] with E value <0.00001. When the results of different databases conflicted with each other, a priority order of nr, Swiss-Prot, KEGG and COG was followed and the best aligned results were used to decide the sequence direction of unigenes and to retrieve proteins with the highest sequence similarity. Further functional classification for all unigenes to understand the distribution of gene functions of the species from the macro level was done using WEGO software [32]. Unigenes which happened to be unaligned to none of the above databases were subjected to ESTScan software [33] for prediction of its coding regions as well as to determine its sequence direction. Fig. 1 represents the bioinformatic pipeline followed for annotation of unigenes.

### Pipeline of Bioinformatic Analysis for SSR Mining, Identification of SSR Bearing Pre-miRNAs and its Possible Targets

**Figure 2:**
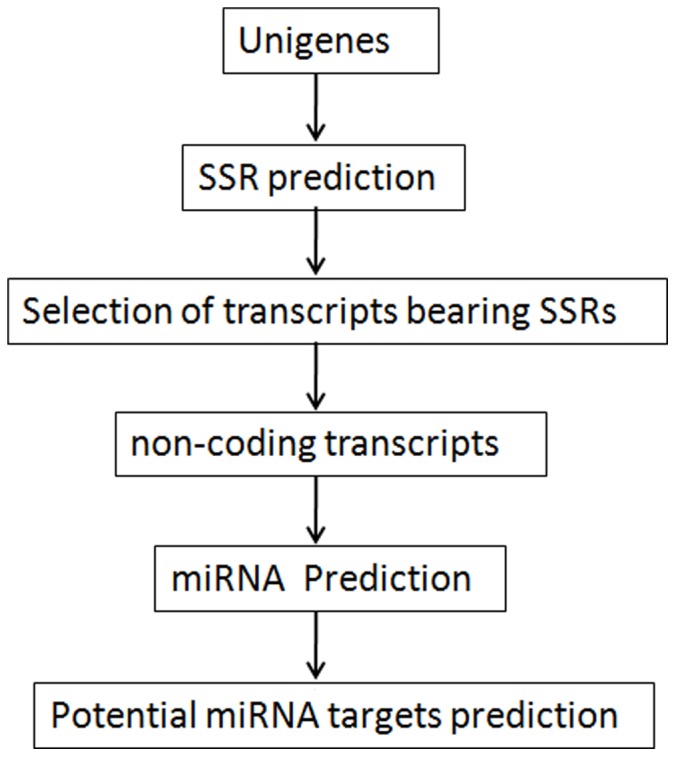
Bioinformatic pipeline followed for identification of SSR bearing pre-miRNAs and its possible targets.

The frequency and distribution of SSRs (dimers, trimers, tetramers, and pentamers) within the unigene sequences were determined using a Perl script - Simple Sequence Repeat Identification Tool (SSRIT) [34] (http://www.gramene.org/db/markers/ssrtool. Accessed 2013 Jan 17). The parameters used included ‘pentamer’ as the maximum motif-length group and the minimum number of repeats allowed was ‘5’ to match SSRs with five or more motif repeats, such as ag-5 (‘agagagagag’). From the identified transcripts bearing SSRs, the ‘unannotated transcripts’ which were considered as non-coding alone were chosen and subjected to miRNA predictions using ‘findMiRNA’programme [35]of Softberry (www.softberry.com. Accessed 2013 Jan 17). The selection criteria adopted for the identification of miRNA candidates were 1) the sequences of predicted precursor miRNA should fold into a hairpin secondary structure that contain the mature miRNA in one arm of the hairpin 2) mature miRNA had less than six mismatches with the opposite arm (miRNA*) 3) the hairpin secondary structure should have a folding energy in the range of≤–32 to –57 kcal/mol 4) the AU content of pre-miRNA should be between 30 and 70% 5) There is no large loop or break in the miRNA sequences [36]–[39]. The secondary structure of RNA was predicted using MFOLD program [40]. In cases, where more than one hairpin stem–loop structure occurred in a single unigene, each of the structures were manually inspected as per the above mentioned selection criteria and the structure with the lowest free energy was selected [41]. The potential plant miRNA targets were analyzed using online available tools like psRNATarget [42]. The transcripts annotated from the transcriptome assembly (BGI) of black pepper was used as target candidates in the user submitted small RNAs/transcripts option of the psRNA target to identify possible targets of miRNAs. Fig. 2 represents the bioinformatic pipeline followed for identification of SSR bearing pre –miRNAs and its possible targets.

## Results

### High Throughput Sequencing and Assembly of Transcripts

**Figure 3:**
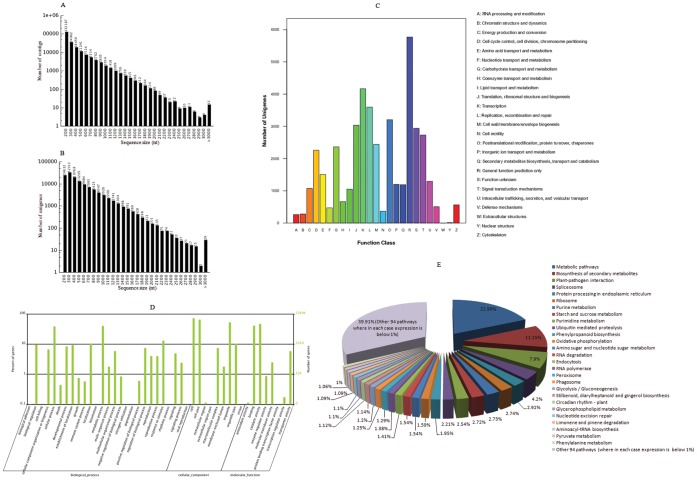
Summary of *de novo* transcriptome sequencing and assembly of black pepper. (A) The length distribution of contigs (B) The length distribution of Unigenes (C) Histogram showing unigene classification based on clusters of orthologous groups (COG) (D) Gene Ontology classification of unigenes (E) KEGG functional classification of unigenes.

To obtain the summary index of transcripts and its expression pattern, we carried out the *de novo* transcriptome illumina sequencing and assembly. Before proceeding, the RNA was subjected to quality check with Agilent 2100 which resulted in a RIN value of 8.10; 28S:18S ratio of 1.9, concentration of 1056 ng/*µ*L and a total mass of 51.744 *µ*g. A total of 55,072,366 raw sequencing reads with a length of 90 bp having a total of 4,956,512,940 nt with 94.24% Q20 percentage were obtained. The raw reads when assembled using Trinity program resulted in a total of 2, 23, 386 contigs with a total length of 59, 024,470 nt and an average length of 264 bp. About 59.17% of contigs occurred in the length range of 100 – 200 nt, 15.65% in 200–300 nt, 8.26% in 300–400 nt, 5.03% in 400–500 nt and contigs with more than or equal to 500 nt accounted for 11.88%. The length distribution of contigs is shown in Fig. 3A. Contigs were joined to create scaffolds and finally sequences without Ns which cannot be extended on either end were generated to obtain 1, 28,157 unigenes with a total length of 57,481,660 nt and an average length of 449 bp. About 70.16% of unigenes occurred in the length range of 100–500 nt, 22.69% in 500–1000 nt, 5.48% in 1000–1500 nt, 1.3% in 1500–2000 nt and contigs with more than or equal to 2000 nt accounted for 0.38%. The length distribution of unigenes is shown in Fig. 3B.

### Functional Categorization of Transcripts

Functional annotations for the assembled unigenes against protein databases like nr, Swiss-prot, KEGG and COG identified a substantial fraction of resourceful genes (Table S1). COG database predicted and classified possible functions for the unigenes as shown in Fig. 3C. COG is a database where orthologous gene products were classified and included a variety of biological processes like RNA processing and modification, chromatin structure and dynamics, energy production and conversion, cell cycle control, amino acid, nucleotide, carbohydrate, coenzyme and lipid transport and metabolism, translation, ribosomal structure and biogenesis, transcription, replication, recombination and repair, motility of cell wall and its biogenesis, posttranslational modification, protein turnover, chaperones, inorganic ion transport and metabolism, secondary metabolites biosynthesis, transport and catabolism, signal transduction mechanisms, intracellular trafficking, secretion, and vesicular transport, defense mechanisms, extracellular and nuclear structures and cytoskeleton. Gene Ontology (GO) is an international standardized gene functional classification system which offers a dynamic-updated controlled vocabulary and a strictly defined concept to comprehensively describe properties of genes and their products in any organism. GO has three ontologies: molecular function, cellular component and biological process. The basic unit of GO is GO-term. Every GO-term belongs to a type of ontology. With nr annotation, Blast2GO program generated GO annotation for the unigenes and WEGO software enabled the subsequent GO functional classification. Based on different kinds of functional categories, the biological process made up majority, followed by cellular component and molecular function. Whereas on the basis of number, higher incidence of unigenes were under cellular component (67,889), followed by biological process (55,276) and molecular function (31,448) as shown in Fig. 3D. Under the biological process, cellular process (12,490), metabolic process (13,072) and response to stimulus (4,237) classes were most prominently represented. The least represented classes include biological adhesion (33), cell killing (1), locomotion (12), nitrogen utilization (3), pigmentation (18), rhythmic process (12) and viral reproduction (20). In cellular component, cell (23,286), cell part (21,068) and organelle (17,133) were more prominent when compared to extracellular region (37), extracellular region part (18) and virion (11). Under molecular function, binding (13,209) and catalytic activity (15,147) occurred more when compared to least represented classes like antioxidant activity (37) and protein binding transcription factor activity (24). KEGG is a bioinformatic resource for linking genomes to life and environment. KEGG records networks of molecular interactions in the cells and variants of them specific to particular organism thereby enable to understand biological functions of genes. About 121 pathways were annotated for all the unigenes according to KEGG functional analysis as shown in Fig. 3E. The major represented was metabolic pathway constituting 6,359 genes (22.99%). The sequence data from this study have been submitted to the NCBI Sequence Read Archive [NCBI: SRA050094 (Study Sample SRS291728)].

### Characterisation of Microsatellite Repeats in Transcripts

**Figure 4:**
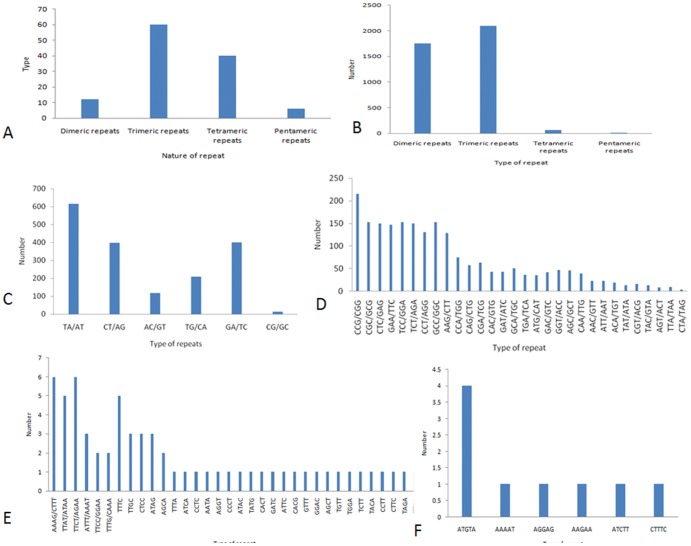
Summary of microsatellite repeats identified in the generated transcriptome. (A) Classification of microsatellites based on different types of motifs (B) Classification of microsatellites based on nucleotide string (C) Characterisation of dinucleotide repeats detected in transcripts (D) Characterisation of trinucleotide repeats detected in transcripts (E) Characterisation of tetranucleotide repeats detected in transcripts (F) Characterisation of pentanucleotide repeats detected in transcripts.

Transcriptome profiling of black pepper revealed the presence of a variety of microsatellite repeats in different forms which were categorized based on (a) type of SSR unit that they possess as di, tri, tetra or penta nucleotides and (b) total number of individual type of SSRs. Out of the total 1, 28,157 unigenes identified, about 2.78% (3,564) possessed microsatellite repeats in their transcript sequences. About 309 transcripts contained more than one type of SSR motif in their sequences. By type, the trimeric repeats constituted the most abundant class possessing 60 kinds of different SSR motifs, which was followed by tetrameric having 40 types, di and pentameric repeats each having 12 and 6 kinds of SSRs respectively, as shown in Fig. 4A. By number 2,091 SSRs were classified as trinucleotide repeats which formed the major class. Dinucleotide (1,750) and tetranucleotide (63) repeats formed the subsequent major classes whereas pentanucleotides (9) were the least represented class as shown in Fig. 4B. Among the dinucleotide repeats detected, TA motif was the most abundant (615, 15.72%), followed by GA/TC (399, 10.21%), CT/AG (397, 10.15%), TG/CA (208, 5.4%), AC/GT (118, 3.02%) and CG (13, 0.33%) as shown in Fig. 4C. The CCG/CGG and AAAG/CTTT; TTCT/AGAA motifs were predominantly represented among tri and tetranucleotide repeats respectively as shown in Fig. 4D and 4E. Five different motif sequence types occurred among the pentanucleotide SSRs of which the number of ATGTA motif (4) was relatively more as shown in Fig. 4F. The average frequencies of SSRs were found to be one SSR per 3.04 MB (2.96×10^−3^ GB) of the *P. nigrum* transcriptome. As per SSRIT software, the frequency of SSRs with five iterations was most abundant (61.26%) (Figure S1). SSRs with six iterations constituted 21.67% which was followed by 7 iterations (9.12%); 8 iterations (3.09%); 9 iterations (2.17%); 10 iterations (1.46%); 11 iterations (1.02%); 12 iterations (0.20%); 13 iterations (0.03%) and 16 iterations (0.03%).

### Identification of ‘SSR Bearing Pre-miRNA Candidates’ and its Potential Targets

Approximately 183 unigenes bearing different types of microsatellite motifs showed no reliable homology in any of the public databases. Hence, these ‘unannotated transcripts’ were analyzed for their possibility to be ‘pre-miRNA candidates’. Totally, 43 different ‘pre-miRNA candidates’ were predicted from the 183 unigenes using findmirna program under stringent filtering conditions. The predicted position of ‘pre-miRNA’ and ‘mature miRNA’ and its corresponding sequences, delta G values and AU content are given in Table 1. A few of the lengthy transcripts produced more than one miRNA candidate from different positions of the same unigene, which were denoted as ‘a’ and ‘b’ of a particular annotated unigene. Among the numerous potential targets observed, those with less than or equal to three mismatches, no mismatches between positions 2 to 6 (maximum1 and 0.5 for G-U) and no mismatch at position 10 and11 from the 5′ end of the small RNA and no more than two consecutive mismatches with MFE ≤–30 kcal/mol, were selected. The predicted targets for the identified ‘candidate miRNAs’, is shown in Table 2 of which targets with mismatch at position 10 or 11 (which is the predicted cleavage site) were completely excluded.

**Table 1:** List of putative ‘miRNA candidates’ identified.

Sl no	Unigene ID	SSR motif	Predicted pre-miRNA position	MFOLD Delta G value	Direction	Predicted mature miRNA position	Mature miRNA sequence	A+U content
1	2414	TCC	184–303	41.8	Plus	230–251	AGCGCGAGUUCGCCUUCGCCGU	31.82
2	2535	CGG	8–127	44.29	Plus	67–88	UGCAGCGGUUUGGGAGGAGGGG	31.82
3	3169	AG	218–337	33.2	Minus	282–302	AUGCCGUCUUUCUGGGGUGAG	42.86
4	11980	AG,GGT	37–156	48.18	Minus	114–135	CCUCCUUCUUCGCCUUCCCCCU	36.36
5	12508	AG,TG	78–197	35.3	Minus	88–108	UCUUGGGGGUUGGGGUGAGAG	38.10
6	25668	TCG	126–245	40.4	Plus	172–293	AAGGCUGUGAUGGACGUUUUUG	54.55
7	26112	CCT,AGG	195–314	39.6	Minus	246–267	GGCACCGACGCCAGGGUGUUGU	31.82
8	30088	CTC	34–153	52.4	Plus	111–132	UUGUCGGAGGUGUCGCCAGCGU	36.36
9	31243	CCG	240–359	37.0	Plus	272–292	GGGAGCUUGUUGGUGAUGGUC	42.86
10	32324	GGC	19–138	50.7	Minus	47–68	CUCUAGCACCUUCUCCACUCCG	40.91
11	45182	ACA	229–348	32.6	Minus	283–304	CAAUACUAUGAGCUUGAAUUGG	63.64
12	49665	AAG	147–266	37.9	Plus	243–263	AUGGGGACAACGCAGGUGUUG	42.86
13	49856a	AT	552–671	47.67	Minus	580–500	CAAAUGAACAAUAUAAUUACG	76.19
14	49856b	AT	51–170	37.18	Minus	56–76	UCCUCCACCGUGUAGCUCAUG	42.86
15	49856c	AT	40–159	34.08	Minus	139–159	GCUGCCUGUGGUAGAAUGCGU	42.86
16	50113a	CCT	270–389	41.3	Plus	307–328	AGGAUGAGUUGAAGAAGUUGGU	59.10
17	50113b	CCT	248–367	40.5	Minus	324–345	CGUCGGUGUCGGCCACCACCAA	31.82
18	50626	GAG	77–196	51.1	Minus	113–134	CCAUCGCUGAGCUCGUAGUAGU	45.46
19	51352	CCG	28–147	42.34	Plus	48–69	UUGCCUCUCCUCUCCCAAAGAC	45.46
20	54811	ACC,CCG	29–148	53.86	Minus	111–132	UCACCCCAGAGGUCCGACUCGU	36.36
21	64214a	CCA	59–178	34.04	Minus	106–127	GUAGCCUUUUCGUGUCUGUGUC	50.00
22	64214b	CCA	28–147	38.3	Minus	85–106	CAGUCGAACAUUCGGGCAAGCG	40.91
23	65213	AG	42–161	37.65	Plus	84–104	UCUCUCUUUGUAAGUUUUCUG	66.67
24	72663a	AAG	70–189	36.1	Minus	96–117	AGAUUGAGCUCCUCUUCUUGGU	54.55
25	72663b	AAG	55–174	38.3	Minus	136–157	CCAUGAAUCCAGACUCGGCUGC	40.91
26	74589	AGG	122–241	36.2	Plus	184–204	GUUGGGCGGGGAGGAGAAGAU	38.10
27	88484	AG	50–169	34.5	Plus	131–151	AUCAUAUCUACUCGCUUCAAA	66.67
28	90461	AAG	130–249	46.07	Minus	138–158	ACUCCUUGGUGGUAGACGCCU	42.86
29	93292	AGG	169–288	32.74	Plus	178–198	UGAUGUUGGCGAGGUGAUGAA	52.38
30	94407	TC	216–335	38.1	Minus	245–265	CGACCAUUGCAUGACCAGGGC	38.10
31	94456	GCA	0–119	50.5	Plus	28–49	GCAUUUUGAUGGCCGAUAUGGU	54.55
32	94870	GGA	0–119	41.8	Plus	45–66	UGGGAAGCAGAGGUAUGGCCGC	36.36
33	95728	CTC	18–137	32.77	Plus	57–77	CCUUUCUUGCCGGUUGGAGGA	42.86
34	98341	TC	29–148	35.8	Minus	126–147	GAGAGAGAUGGGGGAUGUCACC	40.91
35	103545a	CGA	61–180	40.6	Plus	84–105	CUGCUCGUCGUCGGAUGGCGAA	36.36
36	103545b	CGA	89–208	42.9	Plus	112–133	GAAGUUAACUUUUCAACGCCGU	59.10
37	106552	ACG	26–145	45.66	Plus	69–90	GCUUCCACUUUGUCAUCCCCCG	40.91
38	111157	TCG	0–119	47.0	Plus	37–58	GAAGAACUCGUCGUCACCGUCG	40.91
39	118746a	GAG	112–231	44.2	Plus	158–179	CUCGCCGAUUUGAGCGGCAGCG	31.82
40	118746b	GAG	86–205	45.6	Minus	102–123	UCCUCAUCCUGCGGCUGCUCUU	40.91
41	120734a	AG	30–149	47.8	Plus	51–71	UCGAGAUGGAGGGAGGUCUGG	38.10
42	120734b	AG	133–252	50.6	Minus	189–209	UCUCCCUCUCUCUCUACUUCU	52.39
43	122117	TTG	233–352	32.0	Plus	291–312	GGGGAUCCGCCAUGAAAGCUCC	36.36

**Table 2:** List of all the potential targets for the ‘miRNA candidates’.

Sl no	Unigene	Target	MFEvalues(kcal/mol)	Target gene description
	ID	Gene ID		
1	74589	*unigene86414	–41.2	* aRecName: Full = Extensin; AltName: Full = Cell wall hydroxyproline-rich glycoprotein; Flags: Precursor; b Extensin OS = Nicotianatabacum GN = HRGPNT3 PE = 2 SV = 1
2	94407	*unigene98674	–35.6	* aZinc finger, C3HC4 type (RING finger) domain containing protein, expressed [Triticumaestivum];
3	120734	*unigene99044	–36.5	* anuclear transport factor 2 family protein [Arabidopsis lyrata subsp. lyrata] >gi|297310548|gb|EFH40972.1| nuclear transport factor 2 family protein [Arabidopsis lyrata subsp. lyrata]; bRasGTPase-activating protein-binding protein 1 OS = Pongoabelii GN = G3BP1 PE = 2 SV = 1
	120734	unigene124275	–9.6	aPREDICTED: hypothetical protein [Vitisvinifera];
4	11980	unigene34919	–24.9	aRecName: Full = Hexose carrier protein HEX6>gi|467319|gb|AAA79857.1| hexose carrier protein [Ricinuscommunis]; bHexose carrier protein HEX6 OS = Ricinuscommunis GN = HEX6 PE = 2 SV = 1
	11980	unigene19725	–24.9	aRecName: Full = Hexose carrier protein HEX6>gi|467319|gb|AAA79857.1| hexose carrier protein [Ricinuscommunis]; bHexose carrier protein HEX6 OS = Ricinuscommunis GN = HEX6 PE = 2 SV = 1
5	26112	*unigene74589	–54.3	NA
6	30088	*unigene87192	–47.3	NA
7	50113	unigene50112	–24.3	NA
				bCell wall protein DAN4 OS = Saccharomyces cerevisiae (strain ATCC 204508/S288c) GN = DAN4 PE = 2 SV = 1
	50113	*unigene40388	–33	* amtn21-like protein [Populustrichocarpa] >gi|222857140|gb|EEE94687.1| mtn21-like protein [Populustrichocarpa]; bAuxin-induced protein 5NG4 OS = Pinustaeda PE = 2 SV = 1
8	50113	*unigene50112	–53.5	NA
				bCell wall protein DAN4 OS = Saccharomyces cerevisiae (strain ATCC 204508/S288c) GN = DAN4 PE = 2 SV = 1
		*unigene50114	–51.4	* aprotein kinase family protein [Arabidopsis thaliana] >gi|75333775|sp|Q9FFW5.1|PERK8_ARATH RecName: Full = Proline-rich receptor-like protein kinase PERK8; AltName: Full = Proline-rich extensin-like receptor kinase 8; Short = AtPERK8>gi|15983497|gb|AAL11616.1|AF424623_1 AT5g38560/MBB18_10 [Arabidopsis thaliana]; bMucin-2 OS = Homo sapiens GN = MUC2 PE = 1 SV = 2
9	50626	*unigene99763	–41.6	* aARI1 (ARIADNE); protein binding/zinc ion binding [Arabidopsisthaliana] gi|75332017|sp|Q949V6.1|ARI1_ARATH RecName: Full = Probable E3 ubiquitin-protein ligase ARI1; AltName: Full = ARIADNE-like protein ARI1; AltName: Full = Protein ariadnehomolog 1>gi|29125018|emb|CAD52883.1| ARIADNE-like protein ARI1 [Arabidopsis thaliana]; bProbable E3 ubiquitin-protein ligase ARI1 OS = Arabidopsis thaliana GN = ARI1 PE = 2 SV = 1
10	72663	*unigene72664	–44.9	* aAt1g05870 [Arabidopsis thaliana]; bNA
	72663	*unigene90314	–33.9	* aCBL-interacting protein kinase 09 [Vitisvinifera]; bCBL-interacting protein kinase 24 OS = Oryzasativa subsp. japonica GN = CIPK24 PE = 1 SV = 1
11	94870	*unigene26914	–45.1	* aNA; bProtein MTL1 OS = Saccharomyces cerevisiae (strain ATCC 204508/S288c) GN = MTL1 PE = 2 SV = 1
		*unigene25530	–45.1	* aNA; bPutative protein TPRXL OS = Homo sapiens GN = TPRXL PE = 5 SV = 2
		unigene14227	–25.8	NA
12	103545	*unigene103544	–33	NA
13	103545	unigene103544	–24.7	NA
14	106552	*unigene22721	–41.5	* aF-box family protein [Cucumismelo subsp. melo]; bF-box protein SKIP22 OS = Arabidopsis thaliana GN = SKIP22 PE = 1 SV = 1
		unigene16846	–22.8	aOs01g0871350 [Oryzasativa Japonica Group] >gi|255673907|dbj|BAH91398.1| Os01g0871350 [Oryzasativa Japonica Group]; bNA
15	122117	unigene122119	–26.8	aNA; bSerine/arginine repetitive matrix protein 1 OS = Musmusculus GN = Srrm1 PE = 1 SV = 1

a Nr-annotation;

b Swissprot-annotation;

* most probable targets.

## Discussion

Next generation sequencing have been successfully applied in many species other than model organisms like sagebrush [43], sweet potato [44], cucumber [45], lentil [46] and ecologically important tree species like pines [47]. Recently, NGS technology has revolutionized the conventional sequencing platforms and among the available NGS strategies, transcriptome sequencing is noticeable for high-throughput rapid discovery of genes. The current study demonstrates the generation of the first leaf transcriptome of black pepper. The available sequence datasets of black pepper was limited, except for the very recent high-throughput sequencing data on root transcriptome of black pepper [48]. Even though the type of sequencing and methodologies followed differed in root transcriptome profiling, an overall comparison of root transcriptome with data generated by us, showed a wider coverage of transcripts (55,072,366 of 90-bp paired-end raw reads) for leaf transcriptome (Table 3). However, the 10,338 unigenes reported for root transcriptome together with our corresponding 1,28,157unigenes (leaf) can be considered a vastly improved ‘resourceful’ tool for biotechnological improvement of black pepper. Trinity - a reference genome-independent assembler produced a total of 2, 23,386 contigs, which when assembled gave 1, 28,157 unigenes, indicating its efficiency to discover new genes. Trinity is reported to be highly efficient to reconstruct the transcriptome, inclusive of the splicing events and transcripts resulting from duplication events, better than other available *de novo* transcriptome assemblers [28].

**Table 3:** A comparison of high throughput sequencing data from recently reported root transcriptome with our generated leaf transcriptome of black pepper.

	Leaf transcriptome	Root transcriptome
Type of sequencing	illuminaHiSeq™	SOLiD platform
Assembly of transcripts	Trinity	multiple-k method
Total number of reads	55072366 (4.9Gbp)	13300000 (665Mbp)
Number of Contigs	223386	22363
Number of Unigenes	128157	10338
Unigene (Total size) (bp)	28740830	1787600
Predicted proteins	73507	4472
RPKM_Most expressed transcript	29348.92	68250

According to KEGG, the most well represented pathway was metabolic pathway (22.99%), followed by biosynthesis of secondary metabolites (11.15%) and plant pathogen interaction (7.9%) as given in Fig. 3E. The least represented pathway was anthocyanin biosynthesis (0.02%). In the metabolic pathways, the presence of 6,359 unigenes implies the active metabolic processes happening during development of leaf tissues in black pepper. The increase in the number of unigenes in the secondary metabolite category i.e. 3,085 unigenes was not at all surprising as black pepper is rich in significant secondary metabolites like piperine and volatile oils [49]. Piperine, a major constituent of pepper is the trans-isomer of I-piperoylpiperidine and accounts for 90–95% of the total pungency of pepper [50]. Therefore, these observed results strongly suggested that most of the genes involved in different pathways have come out through illumina transcriptome sequencing. About 0.14% of the unigenes did not match with any known genes in the public database and were classified as ‘unannotated transcripts’. They may represent either 3′ or 5′ untranslated regions, non-coding RNAs or sequences not containing a known protein domain and their presence in transcriptome as ‘unannotated’ was not surprising as the available sequence of *P. nigrum* in the public database were very few. Hence, these may likely be categorized as novel species specific genes. Unlike model plants, next generation transcriptome sequencing applied in black pepper facilitated the discovery of handful of useful genes and proved to be a real rapid, efficient and high resolution tool.

### Differential Expression of Transcripts

**Figure 5:**
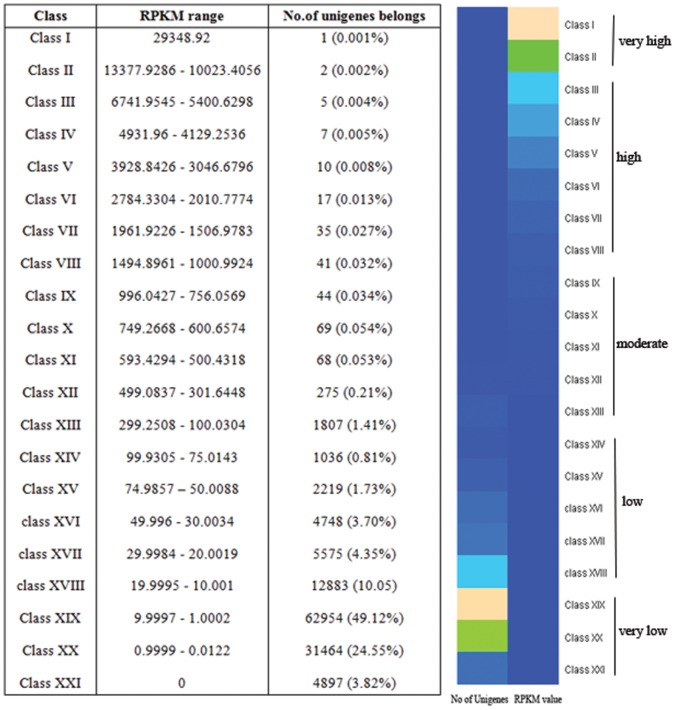
Heat map showing summary of changes in gene expression based on RPKM values.

The RPKM method allowed to study the expression levels of all the unigenes generated. We classified the gene expression in to 21 different classes arbitrarily based on the RPKM values of each transcript and a heat map was generated using 'R script' for the visual comparison of different datasets used in the study as shown in Fig. 5. The largest fraction of transcripts (about 62,954) showed low level of expression in the range of 9.9997 – 1.0002 which belonged to the class XIX. This was followed by 31,464 transcripts of Class XX (0.9999 – 0.0122) and 12,883 transcripts of class XVIII (19.9995 – 10.001). Only a single transcript (unigene 60110_ Uncharacterized mitochondrial protein) was expressed at very high levels with RPKM value of 29348.92. Based on the RPKM values, each of the class was considered as transcripts exhibiting very low/low to moderate to high/very high level of expression. The expression of transcripts were considered to be low if the range of RPKM was below 100, moderate if between 100 to 1,000, high if above 1,000 and very high categories if above 10,000.

### Occurrence, Distribution and Pattern of ‘SSRs in Pre-miRNAs’

In the genomes of many eukaryotes the course of evolution has resulted in a lot of ‘junk DNA’ involving duplications and repeats. Recently, numerous lines of evidence suggest that the genomic distribution of SSRs are nonrandom, and the SSRs located in gene or regulatory regions are reported to have putative functions like their effects on chromatin organization, regulation of gene activity, recombination, DNA replication, cell cycle, mismatch repair system (MMR) etc [51]. The transcriptome survey of black pepper exposed the higher abundance of trinucleotide repeats (53.54%) when compared to di (44.7%); tetra (1.53%) or pentanucleotide repeats (0.23%). This observation was in concurrence with several similar studies in other plants [52]–[54]. Among the SSRs identified, the (AT) repeat was found to be the most abundant (15.72%). This was not surprising as (AT)_n_ repeat motif was suggested as the most frequently occurring microsatellites in plant genomes [55], [56].

**Figure 6:**
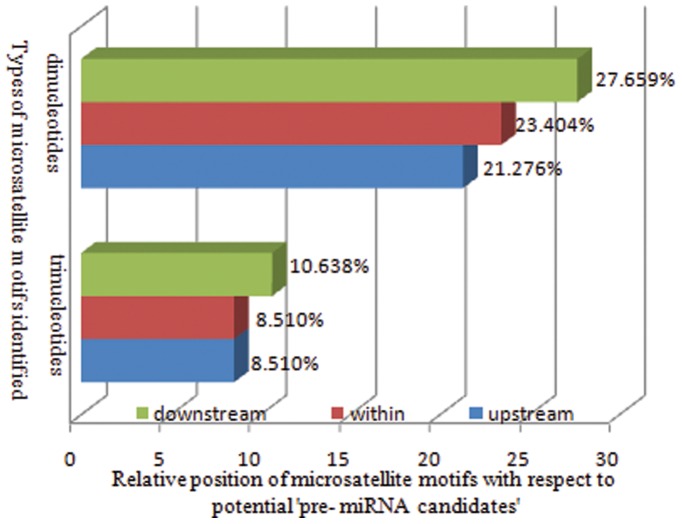
Relative position of microsatellite motifs with respect to potential ‘pre-miRNA candidates’.

Certain repetitive rich regions may give birth to small, but functional RNAs like rasiRNAs, since reports suggest the presence of rasiRNAs in both the sense and antisense orientation of all known repetitive sequence elements, such as long terminal repeat (LTR) and non-LTR retrotransposons, DNA transposons, satellite and microsatellite DNA sequences, complex repeats like the Su (Ste) locus, as well as vaguely characterized repetitive sequence motifs [57]. Such repetitive region associated hcRNAs and rasiRNAs likely play significant regulatory roles [16]. Based on this concept, we assessed the level of incidence of ‘SSRs in the potential pre-miRNA candidates’. Evidence for the ‘presence of SSRs in the miRNA hair-pin precursor’ was well discussed in our previous study [28], but this was limited to a single ‘miRNA candidate’. In the current study the transcriptome of black pepper was analyzed to portray a complete picture regarding the statistical review of the SSRs in the precursors of miRNA candidates. With respect to the 1,28,157 annotated unigenes, about 0.033% constituted ‘SSR bearing pre-miRNA candidates’, whereas with respect to 183 unannotatedunigenes, 23.49% constituted the same. Such an incidence revealing significant number of ‘SSR bearing pre-miRNAs’ in transcripts of black pepper was the first attempt which reflects the potential significance of microsatellites. One of the most intriguing observations was the relative position of SSRs with respect to the position of predicted pre-miRNAs. A slight bias of SSRs towards the downstream region of ‘pre-miRNAs’ was really noticeable. In comparison,the percentage of SSRs occurring within and upstream region of the ‘pre-miRNAs’ was less as illustrated in Fig. 6.

**Figure 7:**
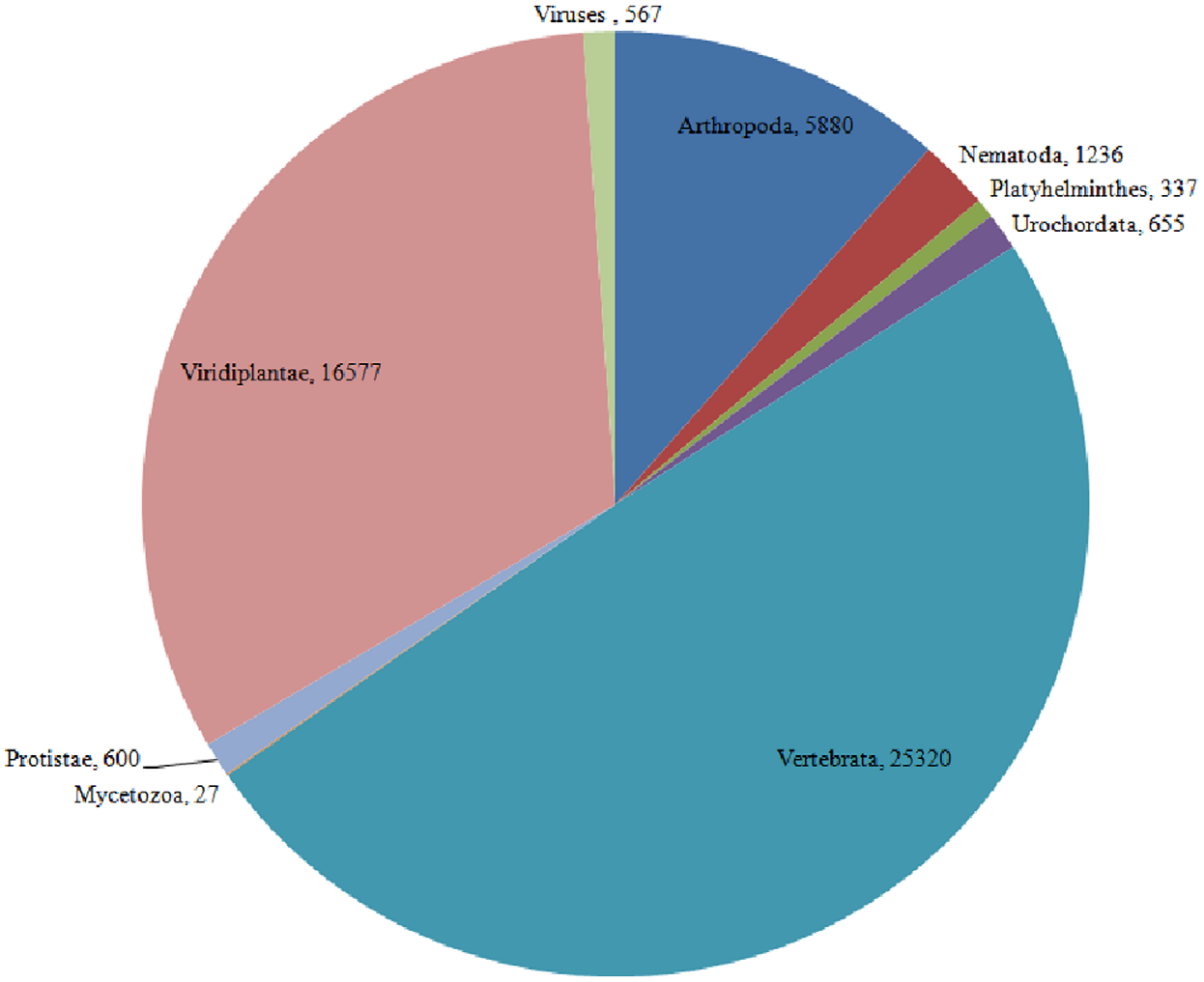
Pie chart showing the relative number of SSR bearing ‘pre-miRNAs’ among different taxa (Viridiplantae, Viruses, Arthropoda, Nematoda, Platyhelminthes, Urochordata, Vertebrata, Mycetozoa and Protistae).

**Figure 8:**
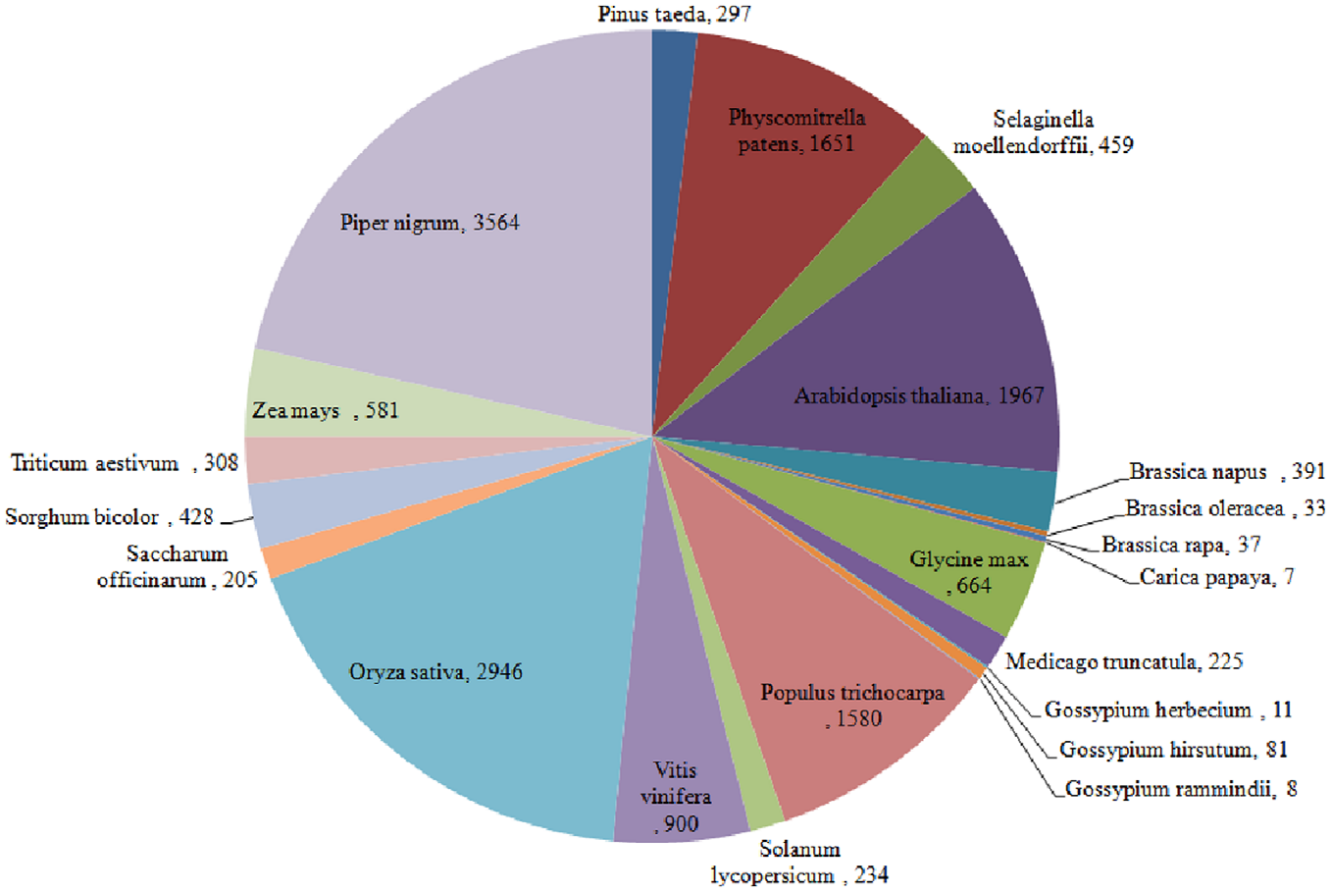
Pie chart showing the relative number of SSR bearing ‘pre-miRNAs’ among different species of Viridiplantae.

**Figure 9:**
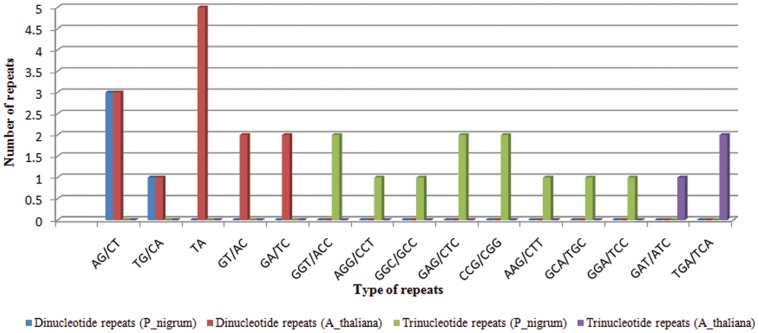
A comparative study between *A. thaliana* and *P. nigrum* on SSRs occurring within the ‘pre-miRNAs’.

An overall comparison of the number of ‘SSR bearing pre-miRNAs’ across different taxa (Fig. 7); and between black pepper and other species of Viridiplantae (Fig. 8) emphasized the biological importance of SSRs occurring in the pre-miRNAs. A more closer and reliable picture regarding the existence of SSRs in pre-miRNAs was portrayed based on a comparison between the model plant –*Arabidopsis thaliana* and *Piper nigrum*. For this, the SSR bearing pre-miRNAs of Arabidopsis, were extracted from the source: ftp://ftp.sanger.ac.uk/pub/mirbase/sequences/12.0/. [27]. The comparison revealed a relative high preference for dinucleotide repeats in pre-miRNAs of *P. nigrum* unlike trinucleotide repeats in pre-miRNAs of *A. thaliana* (Fig. 9). Within the pre-miRNAs of *A. thaliana*, the (AT) repeat type was the most common dinucleotide and (TGA/TCA) was the most common trinucleotide. Whereas (AG/CT) and (TG/CA) were more commonly detected in the ‘pre-miRNAs’ of *P. nigrum* instead of (AT) repeats. Unlike the higher incidence of (AT) repeats detected in the transcriptome SSR survey of *P. nigrum*, its abundance within the ‘pre-miRNAs’ was almost negligible. Thus (AT) repeats may have more possible functions in transcripts rather than ‘pre-miRNAs’. Among the SSRs, (AG/CT) and (TG/CA), were equally distributed within the pre-miRNAs of *A. thaliana* and *P. nigrum*.

More than 30% of the total miRNA candidates detected reliable targets in the transcriptome which enhanced the possibility of the predicted ‘miRNA candidates’ to be ‘true candidates’. A remarkable feature noticed in a few of the potential targets was the presence of ‘tandem usage’ of same amino acids in the miRNA target interaction site. The deduced amino acid in the interaction site revealed ariginine repeats in unigene 99044 (RasGTPase-activating protein-binding protein 1) and glutamate repeats in unigene 90314 (CBL-interacting protein kinase) respectively (Figure S2). This observation emphasized the high significance of transcribed microsatellites in plant genomes. A closer validation of such critical regions in the plant genomes will be a real turnover to the viewpoint that repeat rich regions are just ‘junk and futile’.

### Conclusions

Our attempt to sequence black pepper has contributed towards better understanding of its genomics and updated the current gene resource. The data generated during this study opens up various opportunities for a better understanding of expression patterns and their relation to function and regulation, possible role of transcribed microsatellites in miRNA precursors, as well as geneticmechanismand evolutionary relationships between black pepper and other plants.

## Supporting Information

Figure S1 **Percentage distributions of SSRs based on the no of iterations.**
(TIF)

Figure S2 **Repeat sequences in miRNA target interaction site.**
(TIF)

Table S1 **Table showing complete list of annotated unigenes.**
(XLSX)
