# Supplementary figures and images for: De novo Transcriptome Sequencing Reveals a Considerable Bias in the Incidence of Simple Sequence Repeats towards the Downstream of ‘Pre-miRNAs’ of Black Pepper
Source: PLoS One. 2013 Mar 4;8(3):e56694. doi: 10.1371/journal.pone.0056694 (PMC3587635; doi:10.1371/journal.pone.0056694)

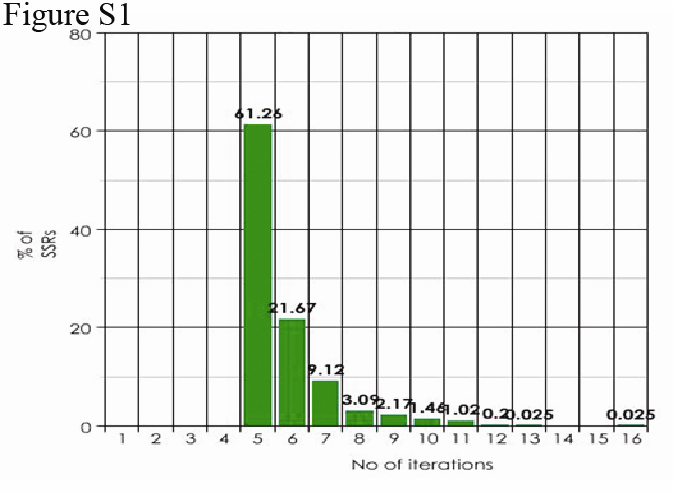

Supplement: Figure S1 — Percentage distributions of SSRs based on the no of iterations. (TIF) [file pone.0056694.s001.tif]

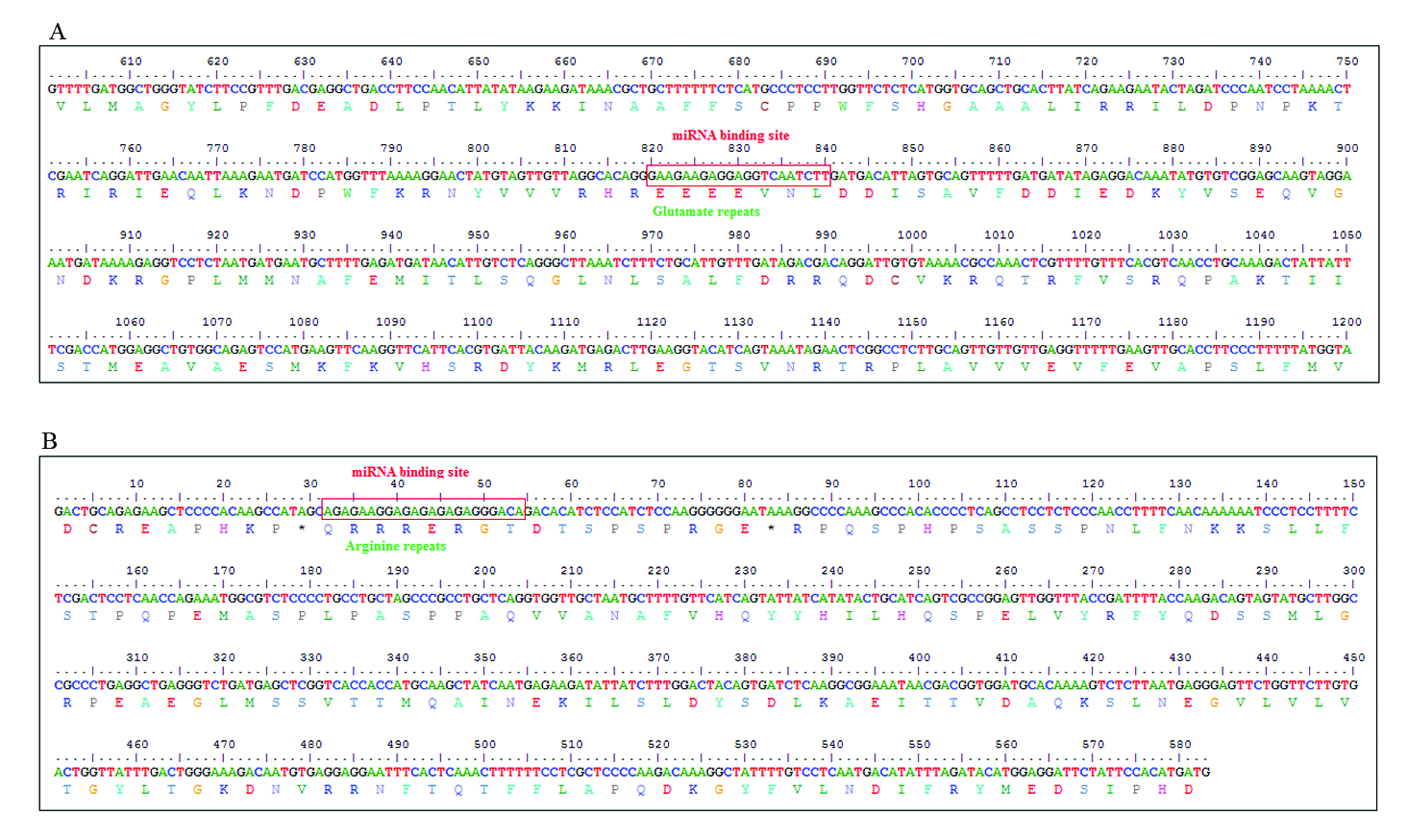

Supplement: Figure S2 — Repeat sequences in miRNA target interaction site. (TIF) [file pone.0056694.s002.tif]
